# Mitochondrial Membrane Remodeling

**DOI:** 10.3389/fbioe.2021.786806

**Published:** 2022-01-04

**Authors:** Ziyun Yang, Liang Wang, Cheng Yang, Shiming Pu, Ziqi Guo, Qiong Wu, Zuping Zhou, Hongxia Zhao

**Affiliations:** ^1^ School of Life Sciences, Guangxi Normal University, Guilin, China; ^2^ Guangxi Universities, Key Laboratory of Stem Cell and Biopharmaceutical Technology, Guangxi Normal University, Guilin, China; ^3^ Research Center for Biomedical Sciences, Guangxi Normal University, Guilin, China; ^4^ National Chengdu Center for Safety Evaluation of Drugs, State Key Laboratory of Biotherapy/Collaborative Innovation Center for Biotherapy, West China Hospital, West China Medical School, Sichuan University, High-Tech Development Zone, Chengdu, China; ^5^ Faculty of Biological and Environmental Sciences, University of Helsinki, Helsinki, Finland

**Keywords:** mitochondrial fusion, mitochondrial fission, mitochondrial dynamics, cristae, crista junctions, membrane curvature, cardiolipin, Mitochondrial disease

## Abstract

Mitochondria are key regulators of many important cellular processes and their dysfunction has been implicated in a large number of human disorders. Importantly, mitochondrial function is tightly linked to their ultrastructure, which possesses an intricate membrane architecture defining specific submitochondrial compartments. In particular, the mitochondrial inner membrane is highly folded into membrane invaginations that are essential for oxidative phosphorylation. Furthermore, mitochondrial membranes are highly dynamic and undergo constant membrane remodeling during mitochondrial fusion and fission. It has remained enigmatic how these membrane curvatures are generated and maintained, and specific factors involved in these processes are largely unknown. This review focuses on the current understanding of the molecular mechanism of mitochondrial membrane architectural organization and factors critical for mitochondrial morphogenesis, as well as their functional link to human diseases.

## Introduction

**FIGURE 1:**
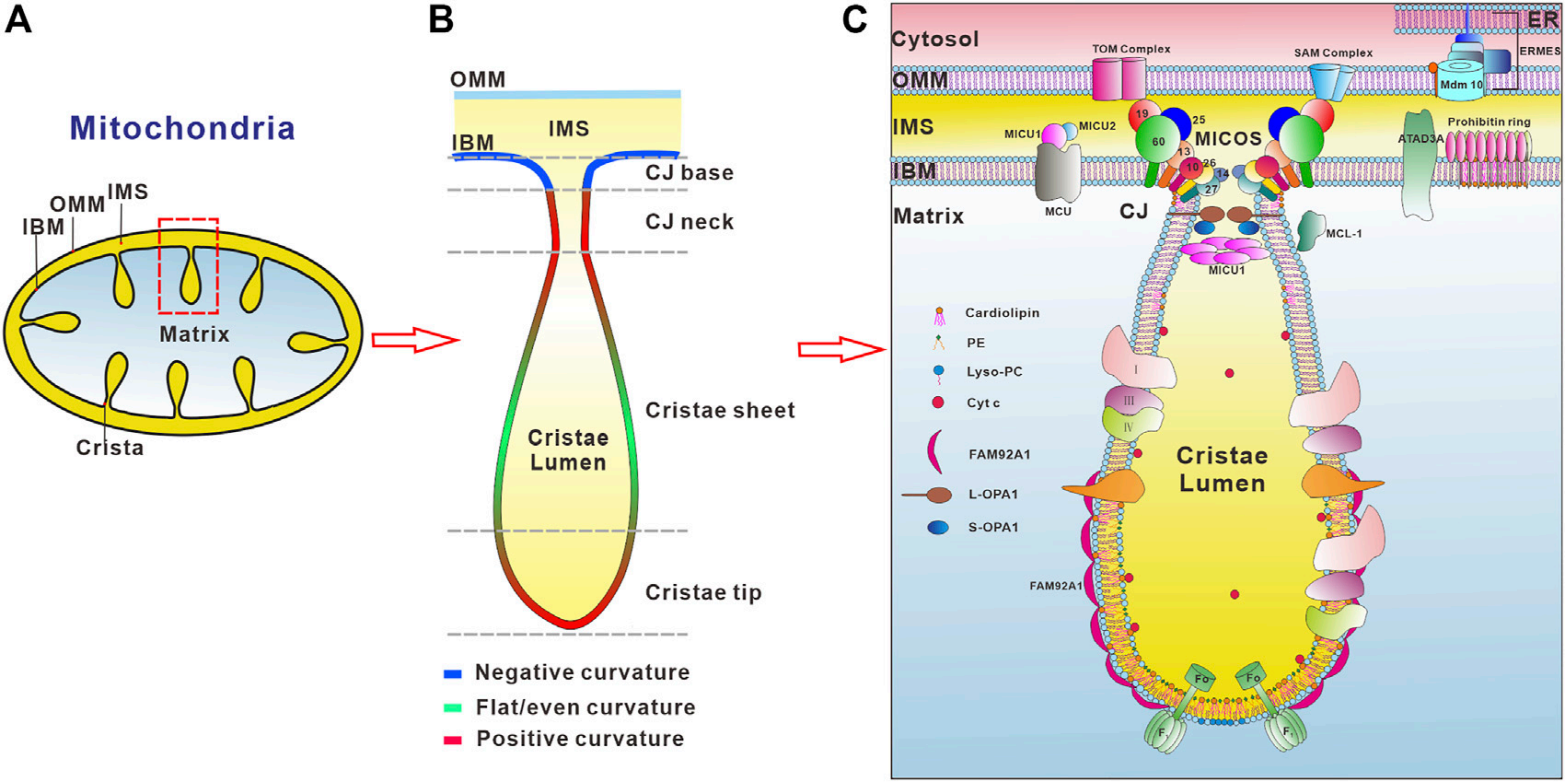
Regulators involved in the organization of mitochondrial inner membrane curvature. **(A)** Schematic presentation of mitochondrial membrane ultrastructure and subcompartments. The outer mitochondrial membrane (OMM) and inner mitochondrial membrane (IMM) delineate two aqueous compartments, the intermembrane space (IMS) (yellow color) between the OMM and the IMM, and the matrix (light blue), which is the innermost compartment. The IMM is further divided into the inner boundary membrane (IBM) and cristae membrane. **(B)** Schematic illustration of membrane curvatures at distinct regions of the cristae membrane. Positive membrane curvature is indicated in red color, negative curvature in blue, and regions with both or no apparent curvature are colored in green. **(C)** Factors involved in the regulation of IMM morphology. F_1_Fo-ATP synthase plays an important role in the formation of positive membrane curvature at the crista tip. The conical cardiolipin and PE reside in the inner leaflet of crista tip membrane while inverted conical lipid such as lysoPC locates in the outer leaflet of the lipid bilayer to maintain the positive membrane curvature at the crista tip. The MICOS complex consists of eight subunits, Mic10, Mic13, Mic14, Mic19, Mic25, MicC26, Mic27 and Mic60, residing in the crista junctions (CJs) (only the numbers are depicted in the figure for the ease of legibility). MICOS is required for the CJs and the contacts between the inner and outer membranes via interaction with the SAM (sorting and assembly machinery) complex. OPA1 is also enriched at the CJs. Interactions between membrane-bound long (L-) and soluble short (S-) forms of OPA1 are required to maintain the width of the CJs. MICU1 exclusively localizes at the crista junctions and binds to the IMM through electrostatic interactions. MICU1 contributes to the structural integrity of the CJ through formation of hexamers. The maintenance of CJ ultrastructure restricts the cytochrome C (Cyt c) inside the crista lumen region. The BAR domain protein FAM92A1 preferentially interacts with the negatively charged phospholipid cardiolipin, and plays crucial role in the formation of positive cristae membrane curvature. In addition, prohibitin ring and ATAD3A localizing in the IBM, and MCL-1 in the IMM and matrix, are also involved in the organization of the IMM.

Mitochondria are double-membrane enclosed organelles in eukaryotic cells essential for cellular metabolism and signaling, housing the key metabolic pathways such as oxidation of nutrients, calcium homeostasis, ROS signaling, and synthesis of heme, steroid hormone, and iron-sulphur clusters. To fulfill these functions, mitochondria form a highly dynamic and motile network that undergoes constant morphology and distribution changes by fusion and fission in response to changes in metabolic requirements, stress, and growth. Huge advances in diverse microscopy technologies and biochemical fractionation of submitochondrial membranes help us to unveil the complex mitochondrial ultrastructure, which possesses the outer (OMM) and inner (IMM) mitochondrial membranes that encapsulate the intermembrane space (IMS) and matrix compartment (Figure 1A). In particular, the mitochondrial inner membrane is folded into numerous invaginations with distinct membrane curvature called cristae (Mannella, 2006) that is functionally and structurally divided into two domains, namely the inner boundary membrane (IBM) and curved cristae membranes. The different domains of the inner mitochondrial membrane have strikingly distinct protein contents, e.g., the respiratory chain complexes and supercomplexes are highly enriched in the cristae membranes, whereas the mitochondrial import and assembly machineries are preferentially found in the inner boundary membrane (Davies et al., 2012; Vogel et al., 2006). Recent studies employing electron microscopy strategies and computer-based reconstruction algorithms have revealed a large variety of cristae morphologies with a mixture of tubular and lamellar segments (Davies et al., 2012; Kühlbrandt, 2015; Mannella, 2006), reflecting a high degree of functional specialization into metabolic micro-compartments. Furthermore, the pleomorphic mitochondrial inner membrane invaginations are connected to the IBM by small circular to narrow slit-shaped openings that are called crista junctions (CJs, Figure 1B) (Mannella, 2006). Based on experimental data a model of cristae membrane organization was proposed (Rabl et al., 2009). In this model, the crista sheets are made up of two leaves of IMM arranged in close apposition, leaving a narrow intermembrane space in between. These sheets are delimitated by tips or rims, in which the lipid bilayer is bending over with a strong positive curvature. At their base, the cristae are connected to the IBM with the membrane exhibiting negative curvature, which is followed by a narrow tubular neck region with highly positive curvature (Figure 1B). The striking breakthrough of super-resolution microscopy techniques, like structured illumination microscopy (SIM) and stimulated emission depletion (STED) microscopy, reveals that both cristae and crista junctions are also highly dynamic, often undergoing membrane-remodeling on a timescale of seconds (Kondadi et al., 2020). Mitochondrial membrane architecture and dynamics are indispensable for energy production, cell division, cell differentiation, and cell death (Wai and Langer, 2016; Dorn, 2019; Sharma et al., 2019; Chan, 2020; Giacomello et al., 2020).

Mitochondria possess not only complex membrane curvature and dynamics The factors involved in membrane but also exhibit defined lipid composition and asymmetric distribution of phospholipids (Horvath and Daum, 2013; Tatsuta et al., 2014; Nielson and Rutter, 2018). Alterations in the phospholipid composition can affect mitochondrial membrane integrity, permeability, and fluidity, and hence the stability and activity of many membrane-associated proteins. Mitochondrial membrane structure, dynamics, and function closely rely on proteins and protein complexes associated with membranes, lipid organization, and the coordinated interplay between proteins and lipids in the mitochondrial membrane. Aberrant cristae ultrastructure, dynamics, and lipid composition cause many devastating human diseases, including neurodegenerative disorders, obesity, diabetes mellitus, muscular dystrophies, cardiomyopathies, and cancer (Chan, 2006; Nunnari and Suomalainen, 2012; Friedman and Nunnari, 2014; Wai and Langer, 2016; Colina-Tenorio et al., 2020; Li et al., 2020; Mukherjee et al., 2021). Although the mitochondrial membrane architecture was discovered with the pioneering work of Palade and Sjöstrand in the 1950s (Palade, 1952; Sjostrand, 1953), the molecular mechanisms underlying the formation of diverse mitochondrial membrane curvature have only been unraveled in part. The factors involved in membrane remodeling and their coordinated interplay to generate, maintain and remold mitochondrial membrane curvature are still largely unknown. This review focuses on the current understanding of molecular mechanisms for the generation of mitochondrial membrane curvature and determinants critical for mitochondrial morphogenesis, as well as human diseases linked to dysfunctional organelles.

## Mitochondrial Membranes and Lipids

Mitochondria feature two phospholipid bilayers with a defined lipid composition. The OMM is smooth, whereas the IMM is extensively folded and highly compartmentalized (Figure 1). The lipid-rich OMM is generally permeable to ions and small, uncharged molecules through pore-forming membrane proteins. Thus, there is no membrane potential across the outer membrane because of its porosity. Larger molecules, especially proteins that are bigger than ∼5,000 Da, have to be imported by special mitochondrial translocases and protein complexes (Schmidt et al., 2010; Wiedemann and Pfanner, 2017). Furthermore, the OMM provides a dynamics platform for cell signaling and tethers with other subcellular compartments to form membrane contact sites, including the endoplasmic reticulum, plasma membrane, lysosomes, peroxisomes, endosomes, and lipid droplets (Scorrano et al., 2019; Huang et al., 2020; Prinz et al., 2020). These membrane contacts have multifunctional roles such as regulation of organelle morphology and dynamics, exchange of ions, lipids, and metabolites across organelles, and signal transduction (Scorrano et al., 2019; Huang et al., 2020; Prinz et al., 2020). In contrast, the IMM contains an extremely high protein content and has much more restricted permeability with an electrochemical membrane potential of ∼120–180 mV (negative inside) across the inner mitochondrial membrane (Logan et al., 2016). Molecules can only get across the IMM with the aid of selective membrane transport proteins. In addition, the IMM contains the protein complexes of electron transport chain responsible for the oxidative phosphorylation system (OXPHOS). The mitochondrial translation machinery, mitoribosomes are also attached to the inner membrane to facilitate the co-translational insertion of the mtDNA-encoded proteins (Brown et al., 2014; Amunts et al., 2015; Greber et al., 2015; Englmeier et al., 2017; Itoh et al., 2021).

The inner and outer membranes of mitochondria define three aqueous subcompartments within the organelle including the intermembrane space, matrix, and crista lumen, each with its distinct role and corresponding protein components (Figure 1C). Between the OMM and IMM is the aqueous sub-compartment IMS. IMS is involved in the import of mitochondrial proteins, the exchange of proteins, lipids, or metal ions between the matrix and the cytosol, initiation of apoptotic cascades, signaling pathways that regulate respiration and metabolic processes, and control of mitochondrial morphogenesis (Edwards et al., 2021). The innermost compartment, surrounded by the inner membrane, is the mitochondrial matrix (Llopis et al., 1998), where DNA replication, transcription, protein biosynthesis and numerous enzymatic reactions occur such as the citric acid cycle and the beta-oxidation of fatty acids. The extended membrane invaginations of the IMM define the crista lumen, which contains large amounts of the small electron carrier protein cytochrome C.

Mitochondria not only have the complex membrane ultrastructure but also specific lipid composition and asymmetrical distribution of proteins and lipids in the OMM and IMM (Figure 1C). In contrast to other cellular membranes, mitochondrial membranes contain only low levels of sterols or sphingolipids (Horvath and Daum, 2013). Furthermore, the lipid composition of the OMM and IMM differs significantly. The OMM is mainly composed of phosphatidylcholine (PC, 54% in rat liver OMM) and phosphatidylethanolamine (PE, 29% in rat liver OMM) (Horvath and Daum, 2013). While phosphatidylinositol is present at a relatively large amount in the OMM (PI, 13% in rat liver OMM), the mitochondria-specific glycerophospholipid cardiolipin is enriched in the IMM (18% in rat liver IMM). In addition, the IMM has different lipid distribution in both leaflets. The monolayer leaflet facing the crista lumen is enriched in PE and cardiolipin with 34 and 18% of IMM phospholipid mass, respectively, while the opposing, positively curved monolayer facing the matrix, contains predominantly phosphatidylcholine (∼80%), with lesser amounts of phosphatidylserine (PS) and phosphatidylinositol (Horvath and Daum, 2013). This asymmetric phospholipid distribution confers stability to a continuously curved cristae membrane, providing an explanation for the high proportion of non-bilayer phospholipids in the IMM. Furthermore, PE and cardiolipin are produced on-site to maintain the cristae membrane phospholipid asymmetry (Tatsuta et al., 2014; Mesmin, 2016; Tatsuta and Langer, 2017). During the process of their synthesis, newly formed PE and cardiolipin segregate to the monolayer leaflet of the cristae membrane facing the matrix. Disruption of PE or cardiolipin synthesis resulted in impaired cristae morphology and oxidative phosphorylation, underlining their indispensable roles in the regulation of mitochondrial function (Claypool and Koehler, 2012; Tasseva et al., 2013; Ren et al., 2014).

The anionic phospholipid cardiolipin is a hallmark lipid of mitochondria, almost exclusively found in the IMM (Horvath and Daum, 2013; Mejia et al., 2014). Cardiolipin is a phospholipid dimer consisting of four acyl chains and two phosphatidyl moieties that are linked to a single glycerol group conferring it with a small polar headgroup. This unique structure of cardiolipin yields a conical shape endowing its curvature sensing/generating abilities (Renner and Weibel, 2011; Beltrán-Heredia et al., 2019; Elías-Wolff et al., 2019). Furthermore, cardiolipin contains tissue-specific acyl chain composition with high content of unsaturated fatty acids that are prone to be oxidized by reactive oxygen species (ROS) generated through the electron transport chain. Cardiolipin plays a central role in mitochondrial metabolism, dynamics, cristae morphogenesis, and signaling (Ren et al., 2014; Dudek, 2017; Panov et al., 2020). Loss of cardiolipin content, alterations in its acyl chain composition, and cardiolipin peroxidation are linked with numerous human diseases in multiple tissues, including Barth syndrome, ischemia, impaired neurogenesis and neuronal dysfunction, cancer, diabetes, and aging (Claypool and Koehler, 2012; Paradies et al., 2019; Panov et al., 2020). The mechanisms that generate, shape, and remodel cristae membranes, however, have only been unraveled in part.

## Remodeling of the Mitochondrial Outer Membrane During Fusion and Fission

**FIGURE 2:**
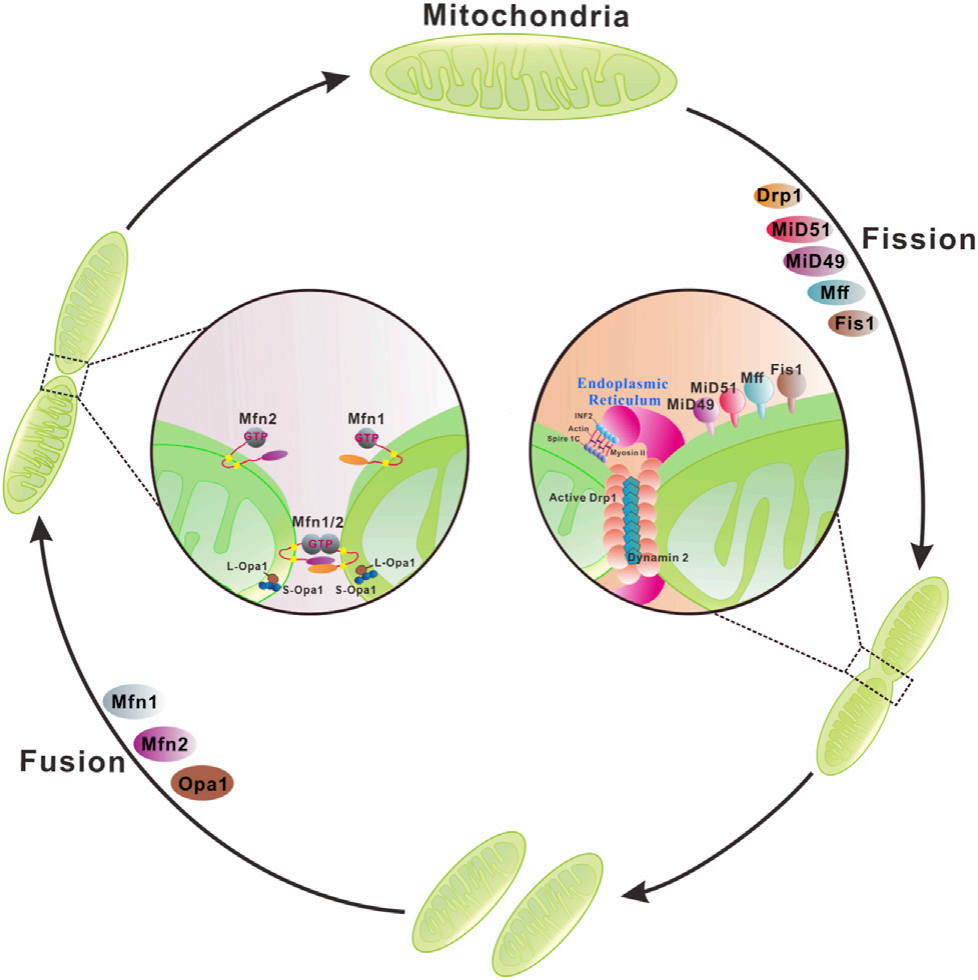
Mitochondrial membrane remodeling during mitochondrial fusion and fission. Mitochondria dynamically change their membrane morphology through coordinated fusion and fission. In mammals, fusion relies on mitofusins 1/2 and optic atrophy protein 1 (OPA1) residing in the outer mitochondrial membrane (OMM) and the inner mitochondrial membrane (IMM), respectively. Mitochondrial fusion is driven by a two-step process with OMM fusion mediated by Mfn1/2 followed by IMM fusion by OPA1. Mfn1/2 forms homo- or hetero-dimers through a *trans* interaction of two opposing OMMs that is essential for mitochondrial fusion and maintenance of mitochondrial morphology. GTP binding or/and hydrolysis induce a conformational change of Mfns, leading to mitochondrial docking and increase of membrane contact sites. Subsequently, GTPase-dependent power stroke catalyzes the OMM fusion. OPA1 has multiple isoforms and can be processed from a long membrane-anchored form (L-OPA1) to a short soluble form (S-OPA1) in the intermembrane space. L-OPA1 and S-OPA1 can form homo- or hetero-dimers/oligomers and interact in *trans* with cardiolipin in the IMM to promote GTP-dependent membrane fusion. Mitochondrial fission involves the organelle pre-constriction followed by scission mediated by dynamin-related protein Drp1. Pre-constriction is facilitated by ER and actin cytoskeleton, specifically to the activities of two actin filament nucleators, formin INF2 and Spire1C12 that reside in the ER and mitochondria, respectively, cooperate to induce actin nucleation and polymerization. Furthermore, the motor protein Myosin II may ensure actin fiber contraction to provide the mechanical force for mitochondrial pre-constriction. The cytosolic Drp1 is recruited to the OMM via multiple transmembrane adaptors MiD51, MiD49, Fis1, and Mff. Drp1 oligomerizes at the ER marked pre-constriction site of OMM, forming a ring-like structure wrapping around mitochondria for further membrane constriction. Dynamin 2 catalyzes the final scission step.

Mitochondrial membranes undergo constant membrane remodeling via coordinated cycles of fission and fusion events, collectively called ‘mitochondrial dynamics’ (Tilokani et al., 2018) (Figure 2). By generating smaller and more discrete mitochondria, fission is essential for cell division and removing the damaged mitochondria by mitophagy. Mitochondrial fission is known to be mediated by cytosolic dynamin-related protein 1 (Drp1) and cofactors that are required for recruitment/activation and assembly of Drp1 on the mitochondrial surface (Cerveny et al., 2007; Mishra and Chan, 2016; Tilokani et al., 2018). Moreover, the endoplasmic reticulum*,* actin polymerization, and calcium influx play important roles in mitochondrial fission (Goldbeter et al., 2013; Chakrabarti et al., 2018). Mitochondrial fusion is the union of two mitochondria for forming a more interconnected mitochondrial network (Gao and Hu, 2021). Hence, fusion allows the replenishment of damaged mitochondrial contents and facilitates intracellular energy distribution. These dynamic transitions are mainly mediated by a small number of evolutionarily conserved, guanosine triphosphatases (GTPases) belonging to the Dynamin family. Two large GTPases constitute the core machinery of mitochondrial fusion, mitofusin 1 (Mfn1) and 2 (Mfn2) residing in the outer membrane, Mgm1 and optic atrophy protein 1 (OPA1) localizing in the inner membrane of yeast and mammal mitochondria, respectively (Mishra and Chan, 2016).

### Mitofusins Are Involved in Outer Mitochondrial Membrane Fusion

Mitochondrial fusion is a multistep process that begins with the juxtaposition and tethering of two adjacent mitochondria via the OMM fusion protein mitofusins (Mfns), Mfn1 (Santetl et al., 2003) and Mfn2 (Merkwirth and Langer, 2008) (Figure 2). Both proteins accumulate at contact areas between two adjacent mitochondria and establish homo or heterotypic complexes leading to mitochondrial fusion (Eura et al., 2003; Hoppins et al., 2011a). Although Mfn1 plays a more active role in the fusion process, Mfn1 and Mfn2 can functionally replace each other. Hence, defects in mitochondrial fusion caused by loss of Mfn1 or Mfn2 can be rescued by overexpressing Mfn2 or Mfn1, respectively (Chen et al., 2005). Despite the functional similarities of Mfn1 and Mfn2 in mitochondrial fusion, some differences between these two molecules have been discovered. Mfn1 has a greater guanosine triphosphate (GTP)-dependent membrane tethering activity and is required for the role of OPA1 in mitochondrial fusion (Cipolat et al., 2004). Mfn2 is not only involved in mitochondrial fusion but is also a key regulator of the mitochondria-endoplasmic reticulum (ER) contact site tethering (De Brito and Scorrano, 2008; Merkwirth and Langer, 2008).

Sequence analysis reveals that the N-terminus of both Mfn1 and Mfn2 contains a gtpase domain followed by a hydrophobic heptad repeat region 1 (HR1) and a second HR2 at the C-terminal region. Two transmembrane (TM) segments of yeast orthologue Fzo1 are predicted to locate between the HR1 and HR2, which contain charged residues allowing the formation of a U-turn in the OMM (Koshiba et al., 2004). This U-turn topology mediates the protrusion of both N- and C-terminus of Mfns into the cytosol. Previous studies suggested that the outer membranes of two opposing mitochondria are tethered by the *trans* interactions of the HR2 and/or gtpase domains of Mfns (Figure 2). GTP binding or/and hydrolysis induce a conformational change of Mfns, leading to mitochondrial docking and an increase of membrane contact sites. Subsequently, GTPase-dependent power stroke catalyzes the OMM fusion. Recently, human Mfns are shown to have only 1 TM that lies between the two HR domains, with the HR2 located in the intermembrane space that is sensitive to oxidative stress (Mattie et al., 2018). This new finding opens a handful of questions concerning the OMM mediators of two opposing mitochondria docking in *trans* necessary for membrane fusion. It is proposed that oligomerization of Mfns on one membrane leaflet or across the opposing membranes may promote and stabilize high membrane curvature, as a prerequisite to undergo membrane fusion (Daumke and Roux, 2017). However, the exact molecular mechanism through which Mfns mediate mitochondrial OMM fusion is still not completely understood. Following the mitofusin mediated outer membrane fusion, dynamin-like gtpase OPA1, the homolog of *S. cerevisiae* Mgm1p, together with cardiolipin in the IMM drive the IMM fusion (Figure 2).

### Drp1 and Adaptors Are Required for Mitochondrial Fission

Mitochondrial fission is a complex process and the dynamin family gtpase Drp1 is a central component of the fission machinery (Figure 2). Drp1 is a cytosolic protein that can translocate to the mitochondrial outer membrane. In contrast to the classical dynamins, Drp1 lacks the specialized pleckstrin-homology domain required for OMM binding (Dar and Pucadyil, 2017; Yonashiro et al., 2009). Instead, Drp1 contains the so-called B-insert region, a loop containing positively charged amino acids at the end of the stalk, that binds adapter proteins on the OMM (Bui and Shaw, 2013). Yeast Dnm1 (a Drp1 orthologue) is recruited to the OMM via the membrane-anchored protein fis1 (Mozdy et al., 2000) and two receptors Mdv1 and Caf4 (Griffin et al., 2005; Tieu and Nunnari, 2000). However, orthologues of Mdv1 and Caf4 have not been identified in mammals. Instead, the mitochondrial fission factor (MFF) and mitochondrial dynamics proteins 49 and 51 (MiD49 and MiD51) act as receptors for Drp1 in mammals (Losó et al., 2013; Otera et al., 2016; Palmer et al., 2011) (Figure 2). On the OMM Drp1 oligomerizes into a ring-like structure wrapping around mitochondria to drive membrane constriction in a GTP-dependent manner (Kraus and Ryan, 2017). However, the intrinsic diameter of helical Drp1 oligomers appears insufficient to circumscribe typical mitochondria with diameters ≥200 nm. Mitochondrial pre-constriction is therefore required for recruitment of Drp1 to the fission sites. The mitochondrial pre-constriction is regulated by endoplasmic reticulum (ER) and actin cytoskeleton (Friedman et al., 2011; Tilokani et al., 2018; Yang and Svitkina, 2019) (Figure 2). At the ER-mitochondrial contact sites, two actin filament nucleators, formin INF2 and Spire1C, that reside on the ER and mitochondria, respectively, cooperate to induce actin nucleation and polymerization required for mitochondrial membrane pre-constriction (Korobova et al., 2013; Manor et al., 2015). Furthermore, the motor protein Myosin II may ensure actin fiber contraction to provide the mechanical force for mitochondrial pre-constriction (Korobova et al., 2014). Interestingly, membrane-bound oligomeric Drp1 induces tubulation of the associated membrane and constricts the membrane in the presence of GTP (Yoon et al., 2001; Mears et al., 2011; Francy et al., 2015). However, the diameter of constricted membrane tubules by Drp1 was 40–60 nm, suggesting that a final membrane scission step is required. Recently, the canonical protein dynamin 2, initially found to play an essential role in endocytic vesicle scission, has been proposed to catalyze this final step (Lee et al., 2016) (Figure 2).

## Key Regulators of Mitochondrial Inner Membrane Curvature

Mitochondria exhibit vast curvature diversity in the architecture of cristae membrane between tissues, organisms, the physiological state, and the developmental stage of cells. The highly folded IMM can be divided into several distinct functional regions including the cristae membrane and the inner boundary membrane that are connected by small circular to slit-like tubular crista junctions (Figures 1B,C). The membrane segment corresponding to the crista junctions also adapts significant curvature, although in this case it is negatively curved on the matrix side and positively curved on the intermembrane space side. Crista junctions are not only important for the cristae architecture, but also critical for regulation of the restricted distribution of proteins, lipids, and soluble metabolites between individual mitochondrial subcompartments (Alkhaja et al., 2012; Harner et al., 2011; Hoppins et al., 2011b; Kondadi et al., 2020; von der Malsburg et al., 2011; Stephan et al., 2020).

Importantly, the diverse membrane curvatures of the cristae and crista junctions can be dynamically remodeled under changes of physiological conditions. Although several models of crista biogenesis and maintenance have been suggested (Rabl et al., 2009; Zick et al., 2009; Harner et al., 2011; Muhleip et al., 2019), the molecular mechanism underlying the formation of cristae membrane curvature still remains poorly understood. Due to recent advances in cryoelectron microscopy (cryo-EM), cryoelectron tomography (cryo-ET), and super-resolution nanoscopy the key molecular players and the molecular details underlying the generation and remodeling of mitochondrial membrane curvature have started to emerge. The factors involved in the mitochondrial membrane remodeling reside in different subcompartments and exhibit crucial, yet different roles in cristae curvature biogenesis and maintenance, including the mitochondrial contact site and cristae organizing system (MICOS) complex, the gtpase optic atrophy type 1 (OPA1), F1Fo- ATP synthase, a BAR domain protein FAM92A1, ATAD3A (atpase family AAA-domain containing protein 3 A), MCL-1, prohibitins, and the calcium uniporter MICU1 (Figure 1C). The local lipid microenvironment is also important in the regulation of the cristae membrane ultrastructure. Furthermore, genetic and physical interactions between the F_1_Fo-ATP synthase, OPA1, and MICOS have been demonstrated (Rabl et al., 2009; Darshi et al., 2011; Janer et al., 2016; Eydt et al., 2017; Rampelt et al., 2017; Quintana-Cabrera et al., 2018), but the functional interplay between these players in cristae development remained largely elusive.

### Crista Tip

The crista tip exhibits a positive membrane curvature on the matrix side and a negative membrane curvature on the crista lumen side. It serves as a dome-shaped cap with an approximately 15 nm radius of curvature that seals the membrane so as to form a barrier between the crista lumen and the matrix (Ikon and Ryan, 2017a). Two key determinants for the membrane curvature at the crista tip is F_1_Fo-ATP synthase complex and local lipid microenvironment, including cardiolipin and PE in the cristae membrane (Figure 1C).

#### F_1_F_o_-ATP Synthase

F_1_F_o_-ATP synthase is a highly conserved enzyme that catalyzes the production of ATP from ADP and Pi. The structure of this multiprotein complex comprises two functional domains: a membrane-spanning subunit (F_o_) and a soluble subunit (F_1_) that generates ATP through the action of a rotational mechanism (Davies et al., 2012). ATP synthesis is powered by a proton transmembrane gradient in mitochondria, which is established by pumping protons to the IMS against their electrochemical gradient across the IMM by the electron transfer chain complexes. The protons passively pass from the IMS to the matrix through Fo, which transfers the stored energy created by the proton electrochemical gradient to F1, causing a conformational change in F1Fo-ATP synthase so that ADP can be phosphorylated to form ATP.

**FIGURE 3:**
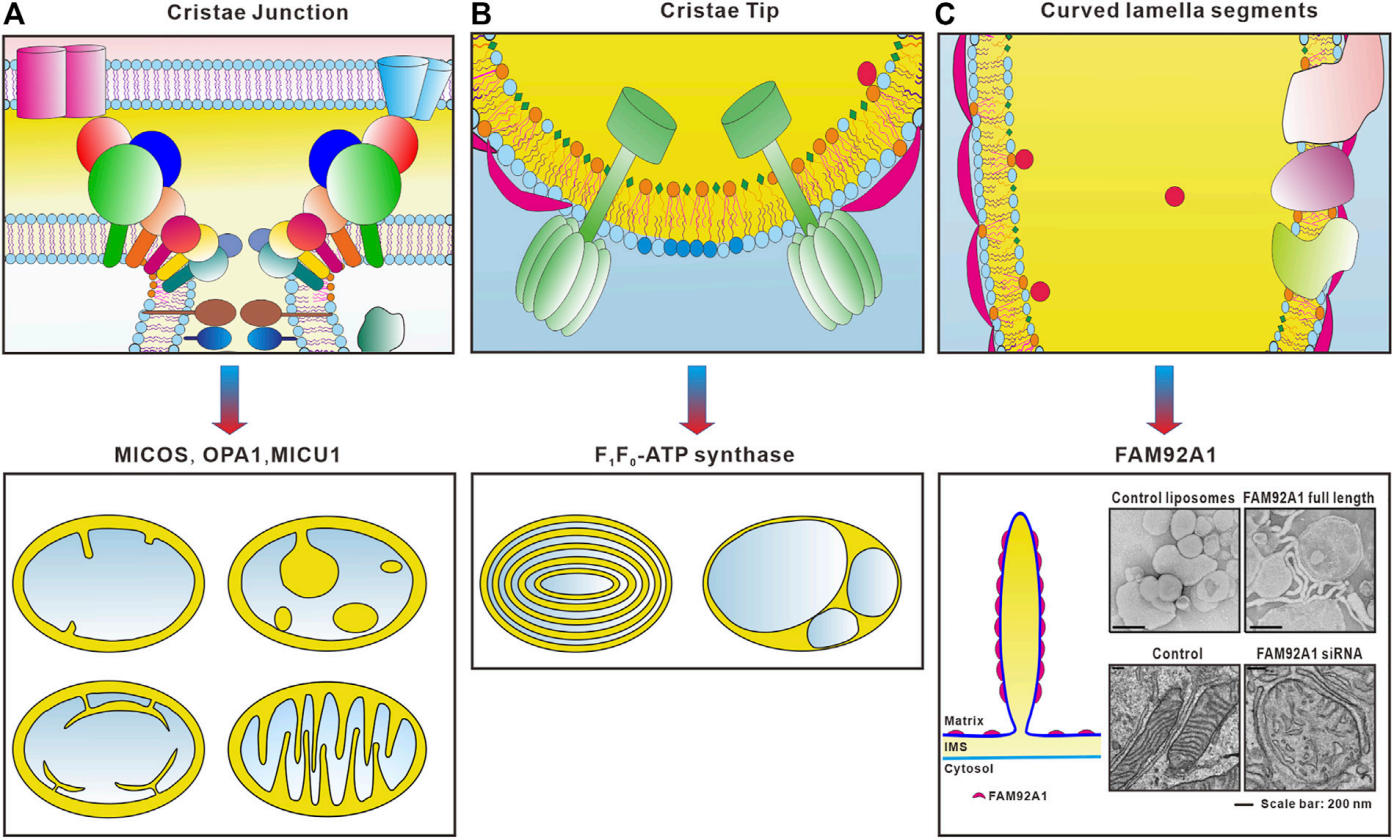
Different regions of cristae display distinct membrane curvature and loss of membrane remodeling factors causes abnormal membrane architecture. **(A)** MICOS complex, OPA1 and MICU1 are important for the formation of crista junctions and depletion or dysfunction of these proteins will cause abnormal membrane architecture, such as disorganized cristae, crista junction widening, decreased crista number or loss of crista. **(B)** F_1_Fo ATP synthase dimers are essential for the membrane curvature of the cristae tip. Dysfunction or loss of F_1_Fo ATP synthase induces concentric onion-like rings or balloon-like cristae morphology. **(C)** FAM92A1 mainly localizes on the lamella segments of cristae and is able to induce membrane tubulation of liposomes with a lipid composition of the IMM (Wang et al., 2019), suggesting that FAM92A1 is involved in generating and maintaining the curved membrane along the lamella regions. Loss of FAM92A1 caused mitochondrial fragmentation, disorganized cristae, decreased crista number and length (Wang et al., 2019).

In 1995, Allen proposed a model suggesting that the association of ATP synthase dimers promotes a distortion of the inner mitochondrial membrane plane (Allen, 1995). Analysis of 2D membrane crystals discloses that a monomeric mitochondrial F_1_Fo-ATP synthase is sufficient to bend a lipid bilayer *in vitro*. This local membrane curvature is likely to be a prerequisite for the formation of ATP synthase dimers and dimer rows, which are necessary for shaping mitochondrial cristae (Jiko et al., 2015). A later study found that deficiency in either subunit *e* or *g* of yeast ATP synthase caused abnormal onion-like or separated cristae corresponding to an uncontrolled remodeling of the inner mitochondrial membrane (Figure 3) (Paumard et al., 2002). This study demonstrated that subunits *e* and *g* of the yeast F_1_F_o_-ATP synthase, which are non-essential components of ATP synthase but required for the dimerization and oligomerization of F_1_F_o_-ATP synthase, are involved in generating mitochondrial membrane curvature. A mutation in the ɑ subunit of yeast ATP synthase causes remarkably aberrant cristae membrane structure while having less effect on ATP production (Kucharczyk et al., 2009). Electron cryo-tomography study with mammalian mitochondria further reveals that the F1Fo-ATP synthase is arranged in rows of dimeric supercomplexes, localized to regions with a pronounced positive curvature such as ridges and rims as well as tubular cristae (Strauss et al., 2008). The structure of the ATP synthase dimer from bovine heart mitochondria shows that the enzyme is overall cone-shaped (Minauro-Sanmiguel et al., 2005). The spontaneous curvature of the mitochondria ATP synthase dimers can introduce positive curvature into the IMM and stabilize the rims of cristae (Paumard et al., 2002; Strauss et al., 2008; Dudkina et al., 2010; Blum et al., 2019). Thereby, these dimer rows are important determinants of membrane bending in the formation of crista tips (Strauss et al., 2008; Davies et al., 2012; Blum et al., 2019; Muhleip et al., 2019).

### Crista Junctions

Crista junction is the site where a given crista extends inward from the IBM, thus it is the interface between the IBM and cristae membrane. A prominent feature of the crista junction is an approximately 90° bend in the membrane, with positive curvature in the IMS side and negative curvature in the matrix side. Although cristae membrane morphology is diverse in size and shape (Zick et al., 2009), crista junctions appear rather uniform. Crista junctions are highly curved tubular openings that typically display inner diameters between 15 and 40 nm that separate cristae membrane invaginations from the surrounding boundary membrane. Several studies discovered that the mitochondrial contact site and cristae organizing system (MICOS) resides within the IMM, predominantly at the crista junction region, functioning in the formation and maintenance of crista junctions. Moreover, dynamin-like gtpase OPA1 and MICU1 are key factors that play fundamental roles in the formation of crista junctions (Figures 1, 3).

#### Mitochondrial Contact Site and Cristae Organizing System Complex

MICOS is a conserved hetero-oligomeric membrane protein complex. All subunits of MICOS are named as Mic'X' where ‘X' represents the approximate molecular weight in kilodalton (kDa) as determined for the yeast homologs of the complex (Kozjak-Pavlovic, 2017; van der Laan et al., 2016; Pfanner et al., 2014; Stephan et al., 2020). The MICOS complex was originally discovered in budding yeast (Harner et al., 2011; Hoppins et al., 2011b; von der Malsburg et al., 2011), an organism in which MICOS has been the most researched. Downregulation of MICOS subunits results in drastic changes in the IMM morphology and leads to reduced crista junction numbers (Figure 3). Consequently, cristae membranes are detached from the IMM and appear as stacks within the mitochondrial matrix underlining the important role of MICOS in the formation of crista junctions (Alkhaja et al., 2012; Harner et al., 2011; Hoppins et al., 2011b; John et al., 2005; von der Malsburg et al., 2011). However, a molecular understanding of how membrane sculpting occurs at the crista junctions is just emerging.

In mammals, eight MICOS subunits have been identified so far (Kozjak-Pavlovic, 2017), namely Mic10 (MINOS1, yeast Mic10), Mic13 (QIL1, yeast Mic12), Mic14 (CHCHD10), Mic19 (CHCHD3, yeast Mic19), Mic25 (CHCHD6, yeast Mic19), Mic26 (Mic23 or APoO, yeast Mic26), Mic27 (APoOL, yeast Mic26), and Mic60 (Mitofilin, yeast Mic60) (John et al., 2005; Darshi et al., 2011; Alkhaja et al., 2012; An et al., 2012; Weber et al., 2013; Guarani et al., 2015; Koob et al., 2015; Genin et al., 2016; Kozjak-Pavlovic, 2017). These subunits form two distinct subcomplexes, Mic10/13/23/27 and Mic60/19/25, bridged together by Mic19 (Friedman et al., 2015; Guarani et al., 2015). However, the exact functions of these two MICOS subcomplexes, particularly how they bend the membrane to the degree observed at crista junctions, are yet to be defined. Recently, the two MICOS subcomplexes are suggested to have different functions in human cells. The Mic60 subcomplex, which is stable in the absence of the Mic10-subcomplex, is critical for the maintenance of crista junctions and the stability of the holo-MICOS complex. In contrast, the Mic10-subcomplex is essential for the formation of lamellar cristae (Stephan et al., 2020). Mic10 and Mic60 are the core components of the MICOS complex, which are highly conserved during evolution, required for the stability of the MICOS complex (Harner et al., 2011; Hoppins et al., 2011b; von der Malsburg et al., 2011). Mic10 can control the spatial distribution of Mic60 and the formation of Mic60 assemblies (Stephan et al., 2020). Depletion of Mic10 or Mic60 caused dramatically altered cristae ultrastructure. Importantly, Mic10 and Mic60 possess membrane-shaping activities (Barbot et al., 2015; Bohnert et al., 2015; Hessenberger et al., 2017; Tarasenko et al., 2017). Purified Mic10 (MINOS1, yeast Mic10) reconstituted into proteoliposomes induces high-degree membrane curvature in model membranes, generating tubules with a diameter between 15 and 25 nm (Barbot et al., 2015). The membrane-shaping activity of Mic10 relies on the formation of homo-oligomers via conserved glycine motifs in the transmembrane segments of Mic10 (Barbot et al., 2015; Bohnert et al., 2015) because loss of the conserved glycine motifs abolishes its membrane-shaping activity both *in vivo* and *in vitro*. While the oligomerization mutants fail to induce curvature in model membranes overexpression of Mic10 in budding yeast promotes the formation of Mic10 oligomers, which leads to a substantial extension and deformation of cristae membranes and crista junctions (Bohnert et al., 2015). A molecular mechanism underlying the process of the IMM sculpting by Mic10 has been proposed, by which Mic10 adopts a wedge-like structure in the lipid bilayer that can generate curved membranes at the crista junction (Barbot et al., 2015), a similar membrane-shaping model demonstrated for the BAR domain family proteins (Mim and Unger, 2012). Recently, Mic10 was found associated with the ATP synthase dimers, which facilitates the formation of ATP synthase oligomers, suggesting that it may link the two major membrane shaping machineries required for the formation of crista tips and crista junctions, respectively (Rampelt et al., 2017).

Mic60 (mitofilin, yeast Fcj1) is anchored in the inner membrane by a single transmembrane segment at the N terminus (Rabl et al., 2009), forming homo-oligomers. Mic60 associates with at least five other MICOS proteins, namely Mic27, Mic26, Mic19, Mic12, and Mic10. Depletion of Mic60, but not of Mic10, results in the absence of all MICOS subunits (Stephan et al., 2020). Furthermore, loss of Mic60 results in loss of crista junctions and the formation of onion ring-like cristae in *Saccharomyces cerevisiae* and mammalian cell lines (John et al., 2005; Li et al., 2016; von der Malsburg et al., 2011). Compared to WT Hela cells, the number of CJs was close to zero in Mic60-KO cells. In contrast, the occurrence of CJs is reduced by about 25% in Mic26-KO cells and by more than 70% in Mic10-, Mic13-, and Mic19-KO cells. Overexpression of Mic60 in yeast cells and Gram-negative bacterium *E. coli* caused additional branching of cristae membranes and formation of plasma membrane invaginations that are reminiscent of mitochondrial cristae, respectively (Rabl et al., 2009; Bohnert et al., 2015). Importantly, Mic60 displays a direct membrane-shaping activity *in vitro*. Purified yeast Mic60 reconstituted into proteoliposomes induces a high degree of membrane curvature, generating long and branched membrane tubules with diameters between 10 and 20 nm (Tarasenko et al., 2017). In addition, purified soluble forms of Mic60 from the thermophilic fungus *Chaetomium thermophilum and Arabidopsis thaliana* lacking the amino-terminal transmembrane domain cause extensive deformation of liposomes (Michaud et al., 2016; Hessenberger et al., 2017). Importantly, Mic60 plays a prominent role in connecting the inner and outer membranes of mitochondria. The major segment of Mic60 localizing in the intermembrane space associates with several outer membrane proteins involved in mitochondrial protein import and assembly, including the protein translocase of the outer membrane (TOM), the sorting and assembly machinery (SAM), porin (VDAC), metaxins 1 and 2, as well as SLC25A46 (yeast Ugo1) involved in mitochondrial dynamics (Bohnert et al., 2012; Darshi et al., 2011; Harner et al., 2011; Hoppins et al., 2011b; von der Malsburg et al., 2011; Ott et al., 2012; Xie et al., 2007; Zerbes et al., 2012). Moreover, the Mic60 subcomplex bridges the MICOS and SAM complexes in the IMM and OMM, respectively. Depletion of mammalian Sam50 impairs the cristae architecture and assembly of respiratory chain complexes, suggesting an intricate interplay between the structural and functional organization of both mitochondrial membrane systems mediated by the SAM and MICOS complexes (Ott et al., 2012). Mic60 also interacts with disrupted-in-schizophrenia 1 (DISC1) (Park et al., 2010), DNAJC11 (Guarani et al., 2015), TMEM11 (Guarani et al., 2015), and OPA1(Barrera et al., 2016). Recently, Mic60 is found to form clusters preferentially localized in the inner membrane at two opposing sides of the mitochondrial tubules with the twisted and helical arrangements (Bohnert et al., 2015). Interestingly, the Mic60 distribution bands are largely independent of the cristae morphology.

Mic13 (Qil1 or C19orf70), a small inner membrane protein with an amino-terminal transmembrane segment and a carboxy-terminal domain facing the intermembrane space, physically interacts with Mic60 and Mic10 (Guarani et al., 2015). Depletion of Mic13 caused onion-like cristae membranes and a complete loss of crista junctions suggesting that Mic13 is strictly required for the formation of crista junctions (Anand et al., 2016). Furthermore, Mic13 facilitates the efficient assemblies of other subunits including Mic10, Mic26, and Mic27 into the MICOS complex (Guarani et al., 2015). Depletion of Mic13 not only leads to reduced biogenesis of the Mic10 subcomplex but also the dissociation of two MICOS subcomplexes Mic60-Mic19-Mic25 and Mic10-Mic13-Mic26-Mic27. Thereby, Mic13 is also required to stabilize the full MICOS complex by holding two subcomplexes together (Anand et al., 2016).

Mic14 (CHCHD10 or C22orf16) encodes a mitochondrial intermembrane space protein that is enriched at crista junctions residing with Mic60/mitofilin, Mic19/CHCHD3, and Mic25/CHCHD6 (Bannwarth et al., 2014; Genin et al., 2016). Expression of Mic14/CHCHD10 mutant alleles leads to abnormal crista structures with loss of crista junctions (Bannwarth et al., 2014), triggered by partial disassembly of the Mic60/Mic19/Mic25/Mic14 complex upon Mic14 mutations. In addition, mutations of Mic14 cause reductions in nucleoid number and nucleoid disorganization (Genin et al., 2016).

Comparably little is known about the peripheral MICOS protein MIC19 (CHCHD3, yeast Mic19 or Aim13), having a coiled-coil-helix-coiled-coil-helix (CHCH) domain that is essential for the import of MIC19 into mitochondria (Darshi et al., 2011, 2012; Modjtahedi et al., 2016). Human MIC19 possesses five cysteine residues, four of which in the CHCH domain form two disulfide bonds (Sakowska et al., 2015). In contrast to human MIC19, yeast Mic19 does not possess a standard twin Cys-X9-Cys motif but has a simplified Cys-X10-Cys motif that also forms a disulfide bond corresponding to the inner disulfide bond of human MIC19 between Cys193 and Cys204. MIC19 appears to be a regulatory subunit that acts as a redox-sensor of the MICOS complex (Sakowska et al., 2015), which is important for the formation of the MICOS complex and regulation of the mitochondrial membrane architecture. However, MIC19 oxidation is dispensable for its integration into the MICOS complex. MIC19 associates directly with Mic60 (Xie et al., 2007). Downregulation of Mic60/Mitofilin or MIC19 results in disorganized mitochondrial cristae, disassembly of MICOS complex, and reduced number of crista junctions (Darshi et al., 2011; von der Malsburg et al., 2011). However, MIC19 (yeast Aim13) deficiency displays less severe effects compared to Mic60 indicating that MIC19 may regulate the complex stability and/or subunit assembly of the complex. Furthermore, MIC19 mediates mitochondrial outer- and inner-membrane contact by directly interacting with mitochondrial outer-membrane protein Sam50 and Mic60 to form the Sam50-Mic19-Mic60 axis (Tang et al., 2020). In contrast, loss of another CHCH-domain containing protein Mic25 (CHCHD6, yeast Mic19 or Aim13) from mammalian mitochondria has only minor effects on MICOS integrity and cristae morphology, but the specific role of this MICOS subunit remains elusive (Tang et al., 2020).

Mic26 *(*Mic23, ApoO) and Mic27 (ApoOL or FAM121A) are apolipoprotein-O-like proteins sharing substantial sequence similarity. Both subunits are required for the maintenance of the cristae ultrastructure and assembly of the F1 subunit into monomeric F_1_F_o_-ATP synthase (Anand et al., 2020). Depletion of Mic26 and Mic27 caused aberrant onion-like crista structures with loss of crista junctions (Weber et al., 2013; Koob et al., 2015). In addition, single and double deletion of Mic26 and Mic27 in human cells lead to more concentric onion-like cristae with loss of crista junctions than any single deletion, demonstrating overlapping roles of Mic26 and Mic27 in the formation of crista junctions. Furthermore, Mic26 and Mic27 are cooperatively required for global integrity and stability of multimeric OXPHOS complexes by modulating the cardiolipin level (Koob et al., 2015; Anand et al., 2020). Unlike the loss of Mic60, Mic10, or Mic13 that resulted in destabilization of either the whole or part of the MICOS subcomplex, Mic26 and Mic27, however, were dispensable for the stability of the remaining subunits of the MICOS complex and their incorporation into higher molecular weight complexes (Anand et al., 2020).

Besides the MICOS-associated OMM proteins involved in protein import and assembly as discussed above, interaction and function links between the MICOS and the F_1_F_o_-ATP synthase have been demonstrated (Rabl et al., 2009; Eydt et al., 2017; Rampelt et al., 2017). A fraction of Mic10 physically interacts with the ATP synthase dimers and overexpression of Mic10 stabilizes the ATP synthase oligomers (Eydt et al., 2017; Rampelt et al., 2017). In contrast, no direct interaction has been demonstrated between Mic60 and any ATP synthase subunits although a pronounced increase of ATP synthase oligomers is observed upon deletion of Mic60 (Rabl et al., 2009). The dimeric F1Fo-ATP synthase, however, can affect the Mic60 distribution (Stephan et al., 2020). Importantly, Mic60 and the F_1_F_O_-ATP synthase subunits e or g act antagonistically to control the oligomerization of F_1_F_O_-ATP synthase (Rabl et al., 2009). Overall, MICOS promotes the formation of crista junctions, whereas the oligomeric F_1_F_o_-ATP synthase is crucial for shaping the crista rims. The interplay between the MICOS complex and F_1_F_o_-ATP synthase highlights a remarkable molecular mechanism by which they cooperatively play critical roles in shaping the inner membrane to regulate the formation of crista junctions and rims.

#### OPA1

Optic atrophia 1 (OPA1), the homolog of *S. cerevisiae* Mgm1p, plays essential roles in mitochondrial dynamics, cristae integrity, respiratory capacity, and mtDNA maintenance (Cipolat et al., 2006; Serrano Cardona and Muñoz Mata, 2013; Patten et al., 2014). Loss of OPA1 causes mitochondrial fragmentation, abnormal cristae architecture and dynamics (Amutha et al., 2004; Hu et al., 2020; Sesaki et al., 2003; Song et al., 2007) (Figure 3), leading to impaired cell proliferation, mitochondrial membrane potential, respiratory capacity (Olichon et al., 2003; Song et al., 2007; Mishra et al., 2014; Patten et al., 2014), and depletion of mtDNA (Chen et al., 2010; Elachouri et al., 2011). Structurally, OPA1 contains an N-terminal mitochondrial targeting sequence (MTS), followed by a transmembrane domain (TM), a coiled-coil domain, a highly conserved gtpase domain, a middle region, and a C-terminal gtpase effector domain (GED). Eight OPA1 variants are present in humans, which are ubiquitously expressed but their levels display great variability in different human tissues (Olichon et al., 2007). Cleavage of the MTS leads to long isoforms (L-OPA1) anchored to the inner mitochondrial membrane by the transmembrane domain, with the other domains exposed to the inner mitochondrial membrane space. Additional cleavage by OMA1 and YME1L proteases at sites S1 (exon 5) or S2 (exon 5b) results in soluble short isoforms (S-OPA1) devoid of the transmembrane segment in the intermembrane spaces (MacVicar and Langer, 2016; Del Dotto et al., 2017, 2018). The long membrane-bound isoforms L-OPA1 are shown to be required for mitochondrial fusion while short forms S-OPA1 facilitate mitochondrial fission (Ishihara et al., 2006; Griparic et al., 2007; Song et al., 2007). Later experiments, however, demonstrate that expression of any OPA1 isoform is able to restore the cristae morphology, preserve the assembly of respiratory complexes and mtDNA content (Del Dotto et al., 2017). The maintenance of mitochondrial network morphology requires at least two OPA1 isoforms with a specific balance of L- and S-forms and an adequate amount of proteins (Del Dotto et al., 2017).

Importantly, OPA1 oligomers tight the crista junctions and maintain a negative crista junction curvature. OPA1 also physically interacts with the MICOS core component MIC60 to stabilize the curvature of crista junctions (Barrera et al., 2016; Glytsou et al., 2016). Mild OPA1 overexpression can inhibit apoptotic crista remodeling and correct altered cristae shape even if MICOS are altered in dysregulated mitochondria, indicating that OPA1 lies upstream of Mic60 in regulating the number and stability of crista junctions (Glytsou et al., 2016). Furthermore, OPA1 and Mic10 antagonistically influence the size and the distribution of Mic60 assemblies (Stephan et al., 2020). OPA1 can stabilize tubular crista junctions in Mic10-KO cells suggesting that OPA1 works in concert with the Mic10-subcomplex to maintain the tubular crista junctions. Additionally, OPA1 associates with the F_1_F_o_-ATP synthase, and its function relies on the oligomerization of ATP synthase (Frezza et al., 2006; Patten et al., 2014; Quintana-Cabrera et al., 2018; Quintana-Cabrera et al., 2021). Both S-OPA1 and S-Mgm1 are able to induce membrane curvature as observed by tubulation of cardiolipin-containing liposomes, revealing the molecular mechanism by which they regulate the IMM morphology (Tadato et al., 2010; Rujiviphat et al., 2015). Recently, electron cryotomography structural studies of the OPA1 homologue Mgm1 on reconstituted membrane tubes show that Mgm1 from *Chaetomium thermophilum* can assemble into a helical filament on positively and negatively curved membranes (Faelber et al., 2019). Consistently, a structural study with S-OPA1 reveals that S-OPA1 can assemble onto membranes in a helical array with a dimer building block, revealing a power stroke membrane remodeling mechanism during mitochondrial inner membrane fusion (Zhang et al., 2020).

#### MICU1

Mitochondrial calcium uniporter (MCU) complex consists of two pore-forming proteins MCU and EMRE (essential MCU regulator). The activity of the MCU-Complex is controlled by MICU1 (mitochondrial calcium uptake 1) and its paralog MICU2 (Stefani et al., 2016). In contrast to the homogeneous distribution of MCU and EMRE at the IMM, MICU1 forms dimer or hexamer via its C-terminal oligomerization site and exclusively localizes at the IBM through electrostatic interaction of its polybasic domain. Loss of MICU1 causes strong widening of the crista junctions suggesting that MICU1 contributes to the structural integrity of the crista junctions (Figure 3). In contrast, knockdown of MCU or EMRE or both has no effect on the morphology of crista junctions. Hence, as a regulator of the MCU complex MICU1 also plays an essential role in the stabilization of crista junctions (Gottschalk et al., 2019).

### Other Factors Involved in the Regulation of Mitochondrial Membrane Curvature

#### Prohibitin

Mitochondrial prohibitins consist of two subunits PHB-1 (32 KDa) and PHB-2 (34 KDa), belonging to the SPFH (stomatin/prohibitin/flotillin/HflKC) family. Proteins in the SPFH family often are membrane-anchored and perform diverse cellular functions in different organelles (Tavernarakis et al., 1999). Within mitochondria, approximately 14 heterodimers composed of PHB1 and PHB2 assemble into a ring-like macromolecular structure at the inner membrane, which is involved in diverse cellular processes, such as maintenance of cristae structure and assembly of the OXPHOS complexes (Ou et al., 2010; Merkwirth et al., 2012). Deletion of PHB2 leads to specific loss of L-OPA1, resulting in an aberrant cristae morphogenesis, suggesting that prohibitin is required for the formation of mitochondrial cristae by stabilizing a long form of the dynamin-like gtpase OPA1. Furthermore, expression of L-OPA1 in PHB2-deficient cells suppresses this defect indicating that impaired OPA1 processing is the primary cellular defect in the absence of prohibitin. Prohibitin can prevent the hydrolysis of L-OPA1 into S-OPA1 thereby enhancing the effect of OPA1 on the morphology of mitochondrial cristae (Merkwirth et al., 2008). In addition, prohibitins genetically interact with genes modulating the biosynthesis of mitochondrial phospholipids, in particular for cardiolipin and PE, suggesting that the prohibitin complex also acts as a membrane organizer affecting the distribution of cardiolipin and PE by clustering them at distinct sites of the IMM (Osman et al., 2009; Hernando-Rodríguez and Artal-Sanz, 2018). Due to the critical roles of cardiolipin and PE in the regulation of cristae membrane curvature, prohibitin is proposed to maintain the proper ultrastructural organization of the IMM. Furthermore, PHB proteins interact with ATAD3 in human cells (He et al., 2012) that has been shown to control cristae structure (Gilquin et al., 2010; Cooper et al., 2017).

#### MCL-1

MCL-1 is a member of the anti-apoptotic BCL-2 family and plays a critical role in the survival of multiple cell lineages. MCL-1 is proteolyzed at the N-terminus to generate three different MCL-1 species, including the full-length (40 kD), two truncated proteins cleaved at Ile 10 (38 kD) or Leu 33 (MW 36 kD). Within mitochondria, MCL-1 proteins with sizes of 40 kD and 38 kD are enriched in the OMM, whereas the 36 kD form localizes to the IMM and matrix, where they perform distinct functions at different mitochondrial subcompartments. The OMM localized MCL-1 proteins exert their anti-apoptotic activity by antagonizing the BAX and BAK activation to maintain mitochondrial integrity. In contrast, the IMM and matrix localized MCL-1 maintains normal IMM architecture through facilitating mitochondrial fusion and promoting the assembly of ATP synthase oligomers (Perciavalle et al., 2012).

#### FAM92A1

MICOS complex and OPA1 are critical for the formation of crista junctions and F_1_F_o_-ATP synthase is essential for the generation of crista tips (Davies et al., 2012; Ding et al., 2015; Friedman et al., 2015; Guarani et al., 2015; Harner et al., 2011; Hoppins et al., 2011b; Hu et al., 2020; van der Laan et al., 2016; Milenkovic and Larsson, 2015; Mühleip et al., 2019; Rabl et al., 2009; Strauss et al., 2008). However, it is yet to be defined how the deeply curved inner membrane invaginations are formed and in particular whether dedicated membrane-shaping proteins are involved in the process. FAM92A1 is a BAR domain protein localizes to the matrix side of the IMM and the majority of FAM92A1 resides in the lamella segments of cristae (Figures 1C, 3C). Loss of FAM92A1 caused a severe disruption to mitochondrial morphology and ultrastructure underlying its critical role in mitochondrial membrane morphogenesis (Wang et al., 2019) (Figure 3). FAM92A1 preferentially interacts with negatively charged phospholipids such as phosphatidylinositol 4,5-bisphosphate and cardiolipin. Importantly, FAM92A1 possesses a membrane-remodeling activity, inducing a high degree of positive membrane curvature *in vitro* suggesting that FAM92A1 is involved in generating and maintaining the curved membrane along the lamella regions (Figure 3). However, the cooperative interplays between FAM92A1 and the other regulators of cristae membrane curvature including MICOS, ATP synthase, and OPA1 remain to be defined.

#### ATAD3A

ATAD3A (atpase family AAA-domain containing protein 3 A) is a nuclear-encoded protein in mitochondria, which is anchored in the IMM by a central transmembrane domain, with the N-terminal region associating with the inner surface of OMM and the C-terminal AAA+ atpase domain residing in the matrix, thus connecting both the inner and outer mitochondrial membranes. ATAD3 is a component of mtDNA nucleoids and plays a role in mtDNA maintenance (Gerhold et al., 2015). In addition, ATAD3A regulates mitochondrial morphology and controls cholesterol trafficking at the ER-mitochondria contact sites (Gilquin et al., 2010; Gerhold et al., 2015; Issop et al., 2015; Baudier, 2018). Loss of ATAD3A causes abnormal cristae morphology and dynamics (Gilquin et al., 2010; Cooper et al., 2017). Moreover, ATAD3A knockout resulted in increased mitochondrial crista-IBM and crista-crista contacts indicating that ATAD3A negatively regulates the membrane contacts. Furthermore, depletion of ATAD3A remarkably reduced the levels of OPA1, Yme1L, and Mic60, suggesting that ATAD3A may cooperate with OPA1, Yme1L, and MICOS to regulate mitochondrial cristae morphology (Hu et al., 2020). The molecular mechanisms underlying the role of ATAD3A in membrane remodeling and association with other membrane-shaping proteins, however, are unknown and warrant further investigation.

#### Mitochondrial Phospholipids

Besides the membrane-remodeling activities of the above-mentioned proteins and protein complexes, membrane lipids and their organization in a lipid bilayer play crucial roles in the generation of membrane curvature. A eukaryotic cell contains more than 1,000 different lipid species that are not homogenously distributed among intracellular membranes, but instead each organelle has a characteristic lipid composition that is required for its proper function. The specific distribution of lipid species across the different cellular membranes/leaflets determines membrane physical properties, such as permeability, membrane curvature, or membrane surface charge, which regulates protein binding and organelle functions. In addition, protein-membrane interaction can cause dynamic rearrangement of lipid and proteins in the membranes, for example, inducing the formation of membrane microdomains and protein oligomerization, consequently modulating protein function, membrane curvature, and dynamics of both protein and lipids (Epand et al., 2007; Zhao et al., 2013; Anand et al., 2020). On the other hand, membrane curvature can control the distribution of membrane lipids and proteins. For example, cardiolipin sorting is observed with increasing membrane curvature (Beltrán-Heredia et al., 2019). Cardiolipin and PE residing in the IMM play critical roles in mitochondrial membrane morphogenesis. The IMM comprises ∼18 and 34% cardiolipin and PE, respectively (Horvath and Daum, 2013). Cardiolipin, an acidic phospholipid with negative charges, plays essential role in the characteristic shape of the mitochondrial inner membrane (Ikon and Ryan, 2017b). Different from the other glycerophospholipids, cardiolipin consists of two phosphatidyl moieties that share a glycerol head group. Thus, the combination of a compact anionic polar head group and four esterified fatty acyl chains results in a distinctly conical lipid molecule (Schlame, 2008). Based on its molecular geometry, cardiolipin is classified as a “non-bilayer” phospholipid. Usually, cardiolipin forms clusters with a certain size by itself or together with another conical lipid PE, promoting negative curvature in biomembranes where headgroups face the concave surface. Due to their specific cone shape, they are segregated into the negatively curved inner monolayer leaflet of the IMM. This lipid asymmetry can reduce membrane tension at the curved region and is required for the generation and maintenance of membrane curvature at the crista tip. Furthermore, the local enrichment of cardiolipin or PE can lower the energetic cost of negative membrane curvature originating from lipid asymmetry between leaflets and distribution of membrane remodeling enzymes, such as F_1_F_o_-ATP synthase and MICOS (Iovine et al., 2021). Genetic disruption of cardiolipin or PE biosynthesis mediates the impairment of cristae morphology, demonstrating the indispensable roles of cardiolipin and PE in the formation of cristae membrane curvature (Gonzalvez et al., 2013; Tasseva et al., 2013). Moreover, cardiolipin interacts with proteins in the IMM regulating their localization and activities. Cardiolipin can directly bind the mitochondrial F_1_F_o_-ATP synthase and the dimer interface to stabilize the structure of the enzyme, promoting the long distance ribbon-like assembly of F_1_F_o_-ATP synthase dimers and affecting the lateral ultrastructural organization of the cristae membrane (Duncan et al., 2016; Mühleip et al., 2019). The phospholipid PE is also a conical-shaped, non-bilayer phospholipid, which is the most autonomously synthesized phospholipid in the mitochondrial membranes. It is synthesized from PS by the enzyme phosphatidylserine decarboxylase (PSD) through the CDP-ethanolamine pathway (Kennedy and Weiss, 1956). Genetic deletion of the PSD enzyme leads to swollen, rounded, and fragmented mitochondria, indicating that PE is critical for mitochondrial morphology (Steenbergen et al., 2005). Several excellent reviews summarize our current understanding of lipid signaling and its scaffolding role (Tatsuta et al., 2014; Aufschnaiter et al., 2017; Dudek, 2017; Nielson and Rutter, 2018; Royes et al., 2020).

Taken together, factors regulating membrane curvature includes lipid composition and asymmetry, nanoscopic scaffolding by oligomerized protein domains, insertion of transmembrane protein domain that have an intrinsic conical or inverted conical shape, or penetration of amphipathic helices like a wedge into one leaflet of a bilayer (Jarsch et al., 2016). The mechanisms that generate membrane curvature and remodel cristae membranes, however, have only been unraveled in part. In particular, we are only beginning to understand how dynamic the inner membrane is and how the various determinants of membrane shape may communicate to generate both cristae architecture and dynamics. The cooperative interplay of the above-mentioned protein and protein complexes in the regulation of mitochondrial membrane architecture and dynamics is not fully understood. Furthermore, mitochondrial lipids such as cardiolipin and PE are emerging as important players in regulating mitochondrial membrane morphology. Whether they modulate functions of the membrane shaping proteins or protein complexes or whether the nonbilayer lipids directly influence the curvature of the IMM by lipid sorting requires further studies. Answering these questions will allow us to understand the basis of human diseases associated with abnormal IMM morphology, which may promote the identification of potential targets for developing therapeutics for mitochondrial diseases.

## Diseases Linked With Abnormal Mitochondrial Membrane Architecture

Mitochondrial membrane composition and architecture are essential for mitochondrial function and characterizing abnormal mitochondrial structural features may provide insight into the underlying pathogenesis of inherited and acquired mitochondrial diseases. Dysregulation of factors involved in mitochondrial membrane morphogenesis, such as defects in relevant protein complexes or phospholipid synthetic enzymes that result in aberrant cristae architecture have been linked to an increasing number of human diseases including neurodegeneration, cardiomyopathies, diabetes mellitus, aging, and cancer (Zong et al., 2016; Li et al., 2020; Mukherjee et al., 2021). For example, mitochondrial ultrastructural defects are prominent in mitochondrial myopathy. So far, the OPA1-associated human disorders are most studied. OPA1 is an important mitochondrial quality control gene and heterozygous mutations in this gene result in dominant optic atrophy (DOA), a disease specifically affecting the retinal ganglion cells and the optic nerve (Alexander et al., 2000; Delettre et al., 2000). Recent studies show that an increasing number of symptoms involving the central, peripheral, and autonomous nervous systems, with considerable variations of the age of onset and severity, has been found in OPA1-related diseases (Chao de la Barca et al., 2016). This variety of phenotypes is attributed to differences in the effects of OPA1 mutations and to the mode of inheritance. Mutations in the mitochondrial ATP synthase also cause severe phenotypes mainly due to deficiency in bioenergetics. The Leigh syndrome is a neurological disorder that has been associated with mutations in the mitochondrial ATP6 gene encoding the Atp6p (or α) subunit of the ATP synthase (Kucharczyk et al., 2009). Interestingly, a mutation T9176G in yeast ATP synthase exhibits anomalies in mitochondrial morphology, indicating that the pathogenicity of T9176G may not be limited to a bioenergetic deficiency but is also linked to the role of the ATP synthase in mitochondrial morphogenesis. Dysfunctions of the MICOS components caused by mutations also result in diverse human diseases and the MICOS subcomplexes are affected in different diseases (Guarani et al., 2016; Gödiker et al., 2018; Russell et al., 2019; Eramo et al., 2020). Expression of a Mic14 (CHCHD10) mutant leads to the loss of MICOS complex assembly accompanied by a change of cristae ultra-structure and ultimately mitochondrial damage (Genin et al., 2018). Mutations in Mic14/CHCHD10 have been associated with frontotemporal dementia–amyotrophic lateral sclerosis (FTD-ALS) clinical spectrum (Genin et al., 2016), and a founder mutation (p.Gly66Val) in the same gene was identified in Finnish families with late-onset spinal motor neuronopathy (SMAJ) (Penttilä et al., 2015). Mutations in CHCHD2, a CHCHD10 homologue, were linked also with neurodegenerative disease, Parkinson’s disease (PD), and FTD/Alzheimer’s disease (Funayama et al., 2015; Zhou et al., 2019). Null mutations in Mic13 (QIL1) in mitochondria cause early-onset fatal encephalopathy along with liver disease (Guarani et al., 2016).

Alterations in lipid metabolism and dysregulated phospholipid levels in mitochondria have been associated with a broad range of acute and chronic pathological conditions. Abnormal lipid profiles were reported for example, in several diseases including Alzheimer’s disease, Parkinson’s disease, and amyotrophic lateral sclerosis (Shamim et al., 2018; Liu et al., 2020). The mitochondrial signature phospholipid cardiolipin is essential for mitochondrial membrane architecture, dynamics, bioenergetics, protein import, mitophagy, and apoptosis (Falabella et al., 2021). Aberrant cardiolipin content, structure, and localization result in impaired neurogenesis and neuronal dysfunction, contributing to aging and the pathogenesis of neurodegenerative diseases (Falabella et al., 2021). In particular, cardiolipin is susceptible to oxidative damage by reactive oxygen species produced through the mitochondrial electron transport chain due to its high composition of unsaturated acyl chains. Uncontrolled cardiolipin oxidation causes conformational changes that affect the physical properties of the IMM and OXPHOS activity, favoring the release of cytochrome c and other apoptotic factors into the cytosol, leading to cell death (Dudek, 2017). Under stress conditions cardiolipin is translocated from the IMM to the OMM, playing a central role in mitophagy (Chu et al., 2013). Furthermore, the defects in the biosynthesis of cardiolipin caused by a mutation in the tafazzin (TAZ) gene in Barth syndrome is clinically manifested by cardiomyopathy, skeletal muscle weakness, lactic acidosis, organic aciduria, and growth delay (Ikon and Ryan, 2017b; Falabella et al., 2021; Zegallai and Hatch, 2021). The crucial roles of cardiolipin in mitochondrial function and human diseases have been summarized in recent reviews (Paradies et al., 2019; Falabella et al., 2021). Fully understanding of the factors and pathways that regulate mitochondrial ultrastructure and mitochondrial lipid metabolism is required to design promising therapeutic strategies for eliminating dysfunctional mitochondria and renewing the pool of healthy organelles that are needed for the treatment of mitochondrial dysfunctional diseases. The link between protein- and lipid-dependent regulation of the inner mitochondrial membrane morphology and diseases has been summarized in recent reviews (Chao de la Barca et al., 2016; Picard et al., 2016; Vincent et al., 2016; Falabella et al., 2021; Mukherjee et al., 2021).
